# Probing entrainment of *Ostreococcus tauri* circadian clock by green and blue light through a mathematical modeling approach

**DOI:** 10.3389/fgene.2015.00065

**Published:** 2015-02-27

**Authors:** Quentin Thommen, Benjamin Pfeuty, Philippe Schatt, Amandine Bijoux, François-Yves Bouget, Marc Lefranc

**Affiliations:** ^1^Laboratoire de Physique, Lasers, Atomes, Molécules, Université Lille 1 Sciences et Technologies, Centre National de la Recherche Scientifique, Unité Mixte de Recherche 8523Villeneuve d'Ascq, France; ^2^Unité Mixte de Recherche 7621, Laboratoire d'Océanographie Microbienne, Observatoire Océanologique de Banyuls, Centre National de la Recherche Scientifique, Université Pierre et Marie Curie (Paris 06), Sorbonne UniversitésBanyuls sur Mer, France

**Keywords:** circadian clock, entrainment, light quality, photoreceptors, *Ostreococcus tauri*

## Abstract

Most organisms anticipate daily environmental variations and orchestrate cellular functions thanks to a circadian clock which entrains robustly to the day/night cycle, despite fluctuations in light intensity due to weather or seasonal variations. Marine organisms are also subjected to fluctuations in light spectral composition as their depth varies, due to differential absorption of different wavelengths by sea water. Studying how light input pathways contribute to circadian clock robustness is therefore important. *Ostreococcus tauri*, a unicellular picoplanktonic marine green alga with low genomic complexity and simple cellular organization, has become a promising model organism for systems biology. Functional and modeling approaches have shown that a core circadian oscillator based on orthologs of Arabidopsis *TOC1* and *CCA1* clock genes accounts for most experimental data acquired under a wide range of conditions. Some evidence points at putative light input pathway(s) consisting of a two-component signaling system (TCS) controlled by the only two histidine kinases (HK) of *O. tauri*. LOV-HK is a blue light photoreceptor under circadian control, that is required for circadian clock function. An involvement of Rhodopsin-HK (Rhod-HK) is also conceivable since rhodopsin photoreceptors mediate blue to green light input in animal circadian clocks. Here, we probe the role of LOV-HK and Rhod-HK in mediating light input to the TOC1-CCA1 oscillator using a mathematical model incorporating the TCS hypothesis. This model agrees with clock gene expression time series representative of multiple environmental conditions in blue or green light, characterizing entrainment by light/dark cycles, free-running in constant light, and resetting. Experimental and theoretical results indicate that both blue and green light can reset *O. tauri* circadian clock. Moreover, our mathematical analysis suggests that Rhod-HK is a blue-green light receptor and drives the clock together with LOV-HK.

## 1. Introduction

Circadian clocks allow most organisms to keep the time of the day and timely orchestrate their biological activities around the 24-h cycle of day and night (Bell-Pedersen et al., 2005). In particular, the daily organization of life is of most importance for photosynthetic organisms, which drastically modify their metabolism between day and night and must therefore anticipate dawn and dusk transitions (Dodd et al., 2005; Graf and Smith, 2011). The major time-keeping cue for circadian clock is the regular alternation of light and darkness. However, light intensity and quality also depend on much less predictable environmental factors, such as foliage shade, cloud cover, sea turbidity, which absorb light in a wavelength-dependent manner. Marine organisms such as free-floating microalgae are particularly exposed to large changes in light intensity and quality depending on their position in the water column, ocean mixing and the time of the day, raising the question of how light sensing mechanisms and circadian clock architecture should be designed to keep time in complex or unforeseen environments.

One widespread strategy to resolve the spectral content of light utilizes several types of photoreceptors, differing in both their photosensory and regulatory properties. Land plants have evolved a number of phytochrome and cryptochrome photoreceptors (Chen et al., 2004), which not only entrain circadian clock (Somers et al., 1998) but also control various metabolic and developmental processes (Spalding and Folta, 2005; McWatters and Devlin, 2011). The importance of light quality in circadian function is moreover evidenced by the fact that the effect of light on the free-running period (FRP) and on clock resetting typically depends on wavelength and intensity (Somers et al., 1998; Covington et al., 2001; Allen et al., 2006). The green alga *Chlamydomonas reinhardtii* also uses various photoreceptors to control diverse aspects of its life (Hegemann, 2008). A blue-light sensitive phototropin controls its sexual cycle (Huang and Beck, 2003) and its metabolism (Im et al., 2006), while two green-light and blue-light sensitive rhodopsins regulate phototaxis (Sineshchekov et al., 2002). A flavin cryptochrome that absorbs a wide range of wavelengths including in blue and red domains (Gorman and Levine, 1965; Beel et al., 2012) is hypothesized to control wavelength-dependent phase resetting of the circadian clock, which is maximal for green and red light in dark-adapted cells or for blue and red light in illuminated cells (Johnson et al., 1991; Kondo et al., 1991). Furthermore, a bimodal blue to UV histidine kinase rhodopsin was recently identified in *C. reinhardtii* (Luck et al., 2012).

The action of photoreceptors is not only specified by their spectral sensitivity but also by their pattern of expression. Indeed, their activity is often regulated in a circadian manner, thereby restricting their action to some temporal window inside the day. This gating effect has been thoroughly studied in the land plant *Arabidopsis*, where regulation of cryptochromes and phytochromes by the circadian clock distributes their expression peaks over the whole day (McWatters et al., 2000; Toth et al., 2001). Although the circadian clock components that regulate photoreceptor activity are relatively well known (Allen et al., 2006), it is still unclear whether clock-regulated light signaling contributes to shape circadian clock function or to control other cellular processes (Oliverio et al., 2007).

The present study investigates the effect of light quality on the circadian clock of the picoalga *O. tauri* through combined experimental and mathematical approaches. This minute size picoalga has a compact genome with little gene redundancy and powerful tools for gene functional analysis including gene targeting by homologous recombination (Corellou et al., 2009; Moulager et al., 2010; Lozano et al., 2014). Genome wide studies of gene expression unraveled orchestrated transcription of biological processes along the day/night cycle (Monnier et al., 2010). Circadian regulation of key cellular functions such as the cell division cycle, were also observed under free running conditions of constant light (Moulager et al., 2007). Functional approaches, based on reverse genetics and biochemical approaches have demonstrated the central role of two orthologs of Arabidopsis TOC1 and CCA1 clock genes, suggesting that the core circadian oscillator of *O. tauri* may consist of a simple two-gene feedback loop (Corellou et al., 2009). This finding was supported further by mathematical modeling studies, which could reproduce experimental expression time-courses with great accuracy (Morant et al., 2010; Thommen et al., 2010, 2012; Troein et al., 2011; Dixon et al., 2014). Remarkably, some of these analyzes showed that the light sensing mechanisms in *O. tauri* is carefully designed so that the clock is robust to daylight intensity fluctuations (Thommen et al., 2010) but flexibly adapts to photoperiod changes (Thommen et al., 2012), allowing it to cope with weather and seasonal variations (Troein et al., 2009; Pfeuty et al., 2012). However, the evidence was indirect and did not specify the light input pathway components. Other theoretical studies were based on a complete mechanistic description, hypothesizing five different light inputs. The resulting mathematical model agreed reasonably well with data obtained in various experimental conditions (Troein et al., 2011; Dixon et al., 2014), however without displaying the robustness properties evidenced in the other studies. Moreover, this model did not take light quality into account. Deciphering the light input pathway of *O. tauri*, and how it responds to different wavelengths is all the more interesting as *O. tauri* is a marine organism and as such is subjected to large fluctuations in both light intensity and quality, to which the clock must have adapted in order to keep time reliably.

Some progress toward this goal was made recently, as new clock actors with light sensing properties have been identified in the genome of *O. tauri* and functionally characterized. Blue-light sensing proteins such as cryptochromes (CRY) (Heijde et al., 2010) and a LOV-domain containing histidine kinase (LOV-HK) (Djouani-Tahri et al., 2011) were shown to be under circadian control and to contribute to the circadian clock function. It is currently not known how the CRY and LOV-HK photoreceptors inform the clock about light status. Bioinformatic analysis provides cues about the possible signal transduction pathway from blue light photoreception by LOV-HK to the core TOC1/CCA1 oscillator. A reasonable hypothesis is that LOV-HK is involved in a two-component signaling cascade (TCS) targeting TOC1. Indeed, *O. tauri* TOC1 belongs to the family of response regulators (RR) which are usually found downstream of a His-to-Asp TCS, and unlike its *Arabidopsis* homologs, TOC1 displays the conserved aspartyl residue required for phosphotransfer (Corellou et al., 2009). Besides a phosphorelay protein (HPT), a homology search limits the putative actors of such a cascade to LOV-HK itself and to the only other histidine kinase in *O. tauri* genome, which contains a rhodopsin domain, Rhodopsin-HK (Rhod-HK). The photosensory properties and circadian functions of Rhod-HK are less clear than those of LOV-HK, which has been thoroughly characterized (Djouani-Tahri et al., 2011). One hypothesis is that, as animal rhodopsins, Rhod-HK mediates green or blue light signal to the clock, since *O. tauri* lacks canonical long wavelength photoreceptors such as the red light sensing phytochromes.

Here we use experimental and mathematical modeling approach to test the hypothesis that a two-component signaling system sensing short (blue) and longer (green) wavelength through LOV-HK and Rhod-HK synchronizes the TOC1–CCA1 central circadian oscillator with the day/night cycle. Blue and green light conditions are obtained by illuminating cells with light from a blue and a green light-emitting diodes (LED), whose spectra are shown in Supplementary Figure 1. Often, mathematical models of gene regulatory networks are validated by merely comparing experimental and theoretical expression profiles. However, this may not always fully constrain the model, especially in *O. tauri*, where it was shown by Thommen et al. (2010) that time courses recorded when the clock is synchronized contain few hints about the light-sensing mechanisms. Therefore, we constrain the model using two additional pieces of information, response to resetting and free-running period under constant light conditions for different intensities of blue or green light. These additional experiments directly probe the light input pathway in non-physiological situations and thus bring independent information. We not only find that the model obtained is compatible with all the data used to inform it, but moreover that it predicts accurately resetting observed under blue or green light in advanced dawn experiments.

Our experimental and mathematical results show that *O. tauri* clock is entrained by green light as well as by blue light, although it is significantly more sensitive to the latter. This suggests that *O. tauri* can keep the time of day in various situations, from deep below the surface in the open sea, where blue prevails, to shallow coastal waters where strong absorption by chlorophyll pigments and organic substances leads to a dominant green (Pelevin and Rutkovskaya, 1977; Prieur and Sathiendranath, 1981; Morel, 1988). Our mathematical model also suggests that besides the already identified blue-light receptor LOV-HK (Djouani-Tahri et al., 2011), Rhod-HK is functional in informing the clock about day/night status and senses blue light, but also green light to a lesser extent. Thus, its absorption spectrum could very similar to that of the blue-light sensing state of the histidine-kinase rhodopsin photoreceptor recently characterized in *Chlamydomonas* (Luck et al., 2012).

## 2. Results

### 2.1. Construction of the mathematical model

The mathematical model used in this work incorporates LOV-HK and Rhod-HK into a previously studied model of the TOC1–CCA1 oscillator (Morant et al., 2010; Thommen et al., 2010, 2012), and comprises 10 differential equations (Equations 1, 2). It describes how the two photoreceptors are circadianly regulated by the core loop and reciprocally, how they modulate the latter depending on light input in the blue and green channels. We follow the same strategy as in our previous works, keeping the model minimal in order to avoid overfitting. In particular, we neglect compartmentalization, delays in transcription or translation, or the detailed kinetics of the luciferase reporters, as we showed before that this was not required to obtain excellent agreement with experimental data (Morant et al., 2010; Thommen et al., 2010, 2012).

**Figure 1:**
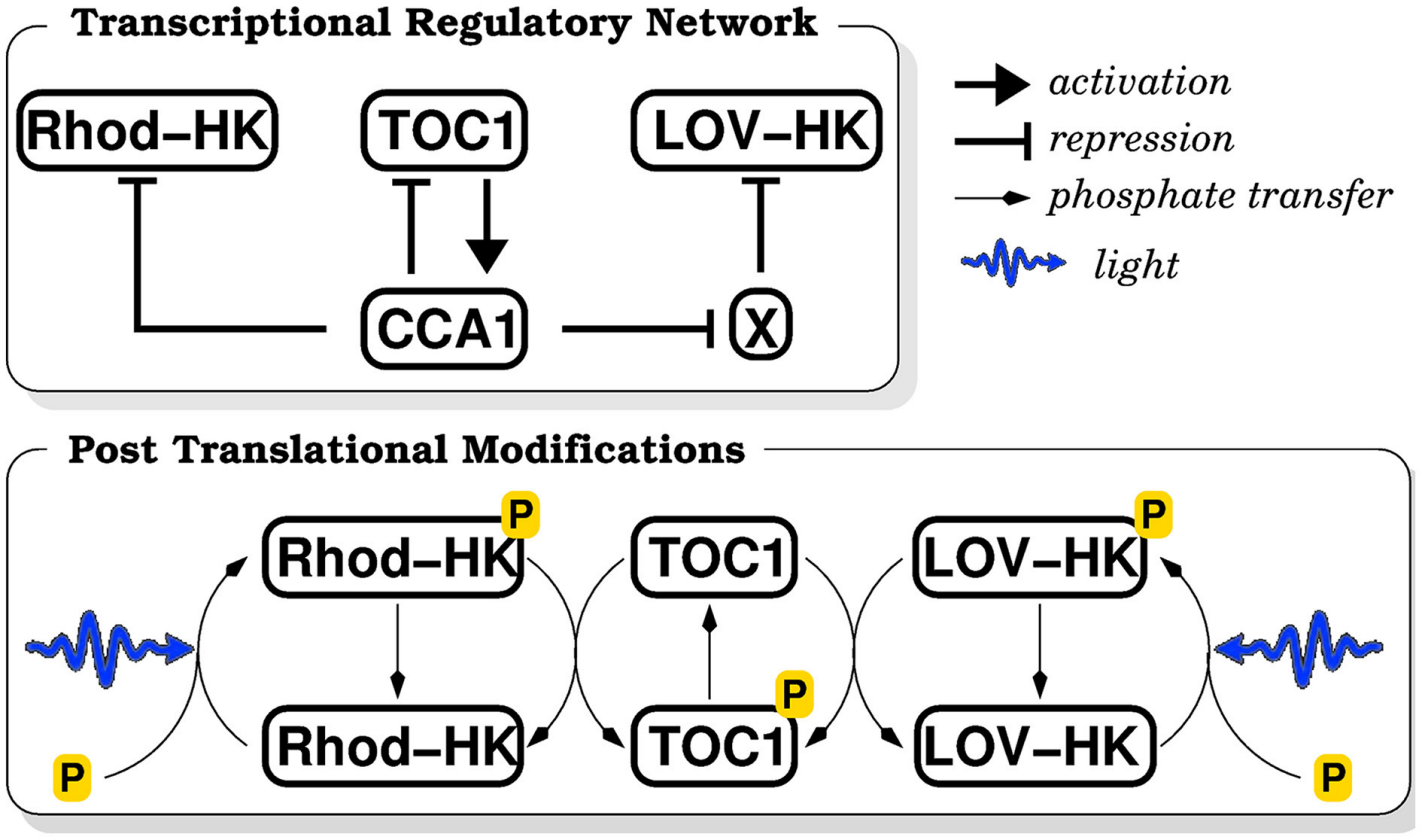
**Schematic diagram of the biochemical reaction network**. The upper panel recapitulates the transcriptional regulations (activation or repression) between molecular actors. The lower panel summarizes the post-translational modifications induced by the phosphate transfer cascade.

As most genes in *O. tauri*, LOV-HK and Rhod-HK mRNAs display strong daily variations, and peak respectively near dawn and dusk in LD 12:12 conditions (12 h of light alternating with 12 h of darkness) (Djouani-Tahri et al., 2011; Pfeuty et al., 2012). Moreover, LOV-HK-Luc luminescent reporter displays rhythms in constant light conditions, showing that LOV-HK is circadianly regulated. The simplest way to take these facts into account is to assume that the two light sensors are directly regulated by the core proteins TOC1 and CCA1, and we accordingly tested different hypotheses. We obtained good adjustment of the model to the data when we hypothesized that CCA1 represses *Rhod-HK* and TOC1 represses *LOV-HK*. However, it may seem strange that TOC1 acts both as a repressor and an activator, even though the transcriptional role of *Arabidopsis* TOC1 has been recently debated (Pruneda-Paz et al., 2009). Therefore, we considered in our model that while Rhod-HK is directly repressed by CCA1, LOV-HK is indirectly activated by it. More precisely, LOV-HK is assumed to be repressed by an unknown actor X, which is in turn repressed by CCA1. This is consistent with the observation that LOV-HK is most expressed in the morning and Rhod-HK in the evening. We insist that while these putative regulations are interesting and reasonable hypotheses, their correctness is not essential for our work, which focuses on the light input to the TOC1–CCA1 oscillator. At this stage, their role is only to generate the correct mRNA time profiles in the mathematical model. The corresponding molecular network is depicted in Figure 1.

In a two-component signaling cascade, upstream histidine kinases (HK) auto-phosphorylate in response to an environmental signal, and then transfer their phosphate group to the downstream response regulator (RR), directly or indirectly via a histidine phosphotransferase (HPT) protein. Here we assume that LOV-HK and Rhod-HK auto-phosphorylate in response to both blue and green light intensities, with efficiencies that are adjustable model parameters. Since the spectral response of Rhod-HK has not been measured yet, this may allow us to determine whether Rhod-HK is more a blue or green light receptor. An HPT protein has been identified in *O. tauri* genome. We assume that HPT integrates the phosphorylation states of LOV-HK and Rhod-HK to control the TOC1 phosphorylation level, which in turn determines TOC1 stability and transcriptional efficacy as activator of CCA1. It has been shown that such TCS pathways can display an ultrasensitive or even bistable response to their input (Igoshin et al., 2008). Accordingly, we assume that TOC1 phosphorylation is characterized by a cooperative response integrating the light intensities perceived by LOV-HK and Rhod-HK, with a Hill coefficient of 4 (Materials and Methods). The main kinetic constants characterizing this response are the sensitivities of LOV-HK and Rhod-HK to blue, green, and white light, their saturation intensities, as well as the variation of the degradation rate and the transcriptional activity of TOC1 between the phosphorylated and non-phosphorylated states.

**Figure 2:**
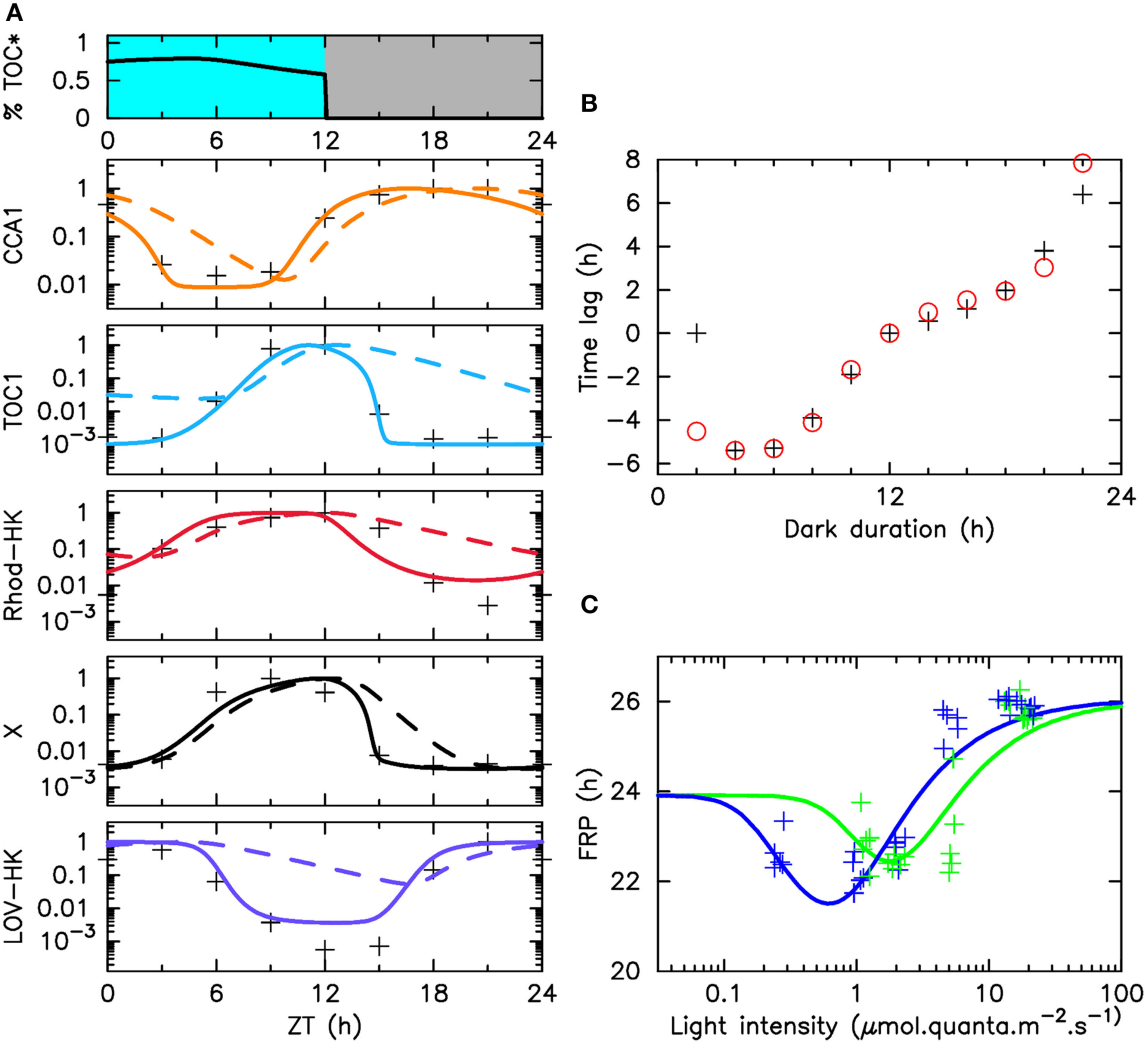
**Adjustment of the model to various experimental data sets**. **(A)** The top panel displays the illumination pattern and the fraction of phosphorylated TOC1. The other panels display, for each molecular actor: the experimental mRNA time profiles under LD 12:12 entrainment (crosses), numerical mRNA time profiles (solid lines) and protein time profiles (dashed lines), in logarithmic scale. **(B)** Phase shifts in resetting experiments as a function of the duration of the last dark period. Phase shifts are obtained as the difference in asymptotic phase with respect to the experiment where this duration is 12 h. Black crosses (resp., red circles) indicate experimental measures (resp., numerical estimates). Raw data are shown in Supplementary Figure 3). In both **(A,B)**, cells are illuminated with the light of a fluorescent tube going through a moonlight blue filter. **(C)** Free running period (FRP) as a function of light intensity in green light (green line) or blue light (blue line). Crosses (resp., solid lines) indicate experimental measures (resp., numerical estimates). Raw data are displayed in Supplementary Figure 4.

Free-running period and resetting measured on luminescent reporter lines under green light indicate that the circadian clock of *O. tauri* is sensitive to green light (see Figure 2). Since green light is not used for photosynthesis, this suggests that this wavelength is perceived through a photoreceptor. Bionformatic analyzes of *O. tauri* genome identified only Rhodopsin as a putative green light sensing domain. However it spectral sensitivity is not known and in particular whether it is more sensitive to blue or green light. Also, there is a small overlap between the LOV-HK absorption spectrum and the green LED emission spectrum (Supplementary Figure 1) so that it cannot be ruled out that green light sensing occurs through LOV-HK. One goal of the modeling approach is clarify these questions.

Combining the description of the circadian regulations and of the TCS described above, we obtain a mathematical model where TOC1 degradation rate and transcriptional activity depends on blue and green light intensities, as well as on the levels of LOV-HK and Rhod-HK, which are themselves regulated by CCA1, the latter being involved in a two-gene negative feedback loop with TOC1. This mathematical model is defined by Equation (1) (Materials and Methods).

### 2.2. Adjustment of the model to experimental data

The mathematical model described in Section 2.1 was simultaneously adjusted to three types of experimental data bringing independent information: (1) mRNA time profiles in LD 12:12 for the four involved molecular actors; (3) measured values of the phase shifts resulting from resetting induced by advanced or delayed dawn, in different light qualities; (3) measured values of the FRP under different intensities of blue or green light. The best-fitting parameter set is given in Tables 1–3. The results of this adjustment procedure, which we detail below, are shown in Figure 2.

**Table 1:** **Best-fitting parameter set for the transcriptional-translation network described by Model (1)**.

**Parameter**		***CCA1***	***TOC1***	***Rhod-HK***	***X***	***LOV-HK***
λ_*Y*_	(nM · h^−1^)	22.58	10.64E+03	5.94E+03	2.20E+03	18.1
μ_*Y*_	(nM · h^−1^)	1.30	75.70	17.91	76.47	0.144
*T*_*Y*_	(nM)	81.12	88.04	10.2E+03	13.2E+03	10.86
*V*_*M*_*Y*__	(nM · h^−1^)	5.83	3.73E+03	18.4E+03	18.9+03	30.71
*K*_*M*_*Y*__	(nM)	1.05	445	895	169	7.17
β_*Y*_	(h^−1^)	1.57	0.129	1.03	10.86	3.60
*V*_*P*_*Y*__	(nM · h^−1^)	1.07	22.64	266	1.89E+03	7.80
*K*_*P*_*Y*__	(nM)	108	5.40E+03	7.79E+03	183E+06	536

**Table 2:** **Best-fitting parameter set for the light input pathway**.

**Parameter**		**Value**
*r*_δ_		0.196
*r*_*Thr*_		0.913
σ_*R*_	(nM^−1^)	5.43E-04
σ_*L*_	(nM^−1^)	0.108
*I*_*R*_0__	(μ mol.quanta.m^−2^.s^−1^)	0.32
*I*_*L*_0__	(μ mol.quanta.m^−2^.s^−1^)	0.33

**Table 3:** **Best-fitting set of intensities *I*_*L*_ (resp., *I*_*R*_) perceived by LOV-HK (resp., Rhod-HK) for various sources of light**.

		**Green LED**	**Blue LED**	**Fluorescent tube**
*I*_*L*_	(μ mol.quanta.m^−2^.s^−1^)	0.22	1.07	0.57
*I*_*R*_	(μ mol.quanta.m^−2^.s^−1^)	0.21	0.36	0.31

We showed in a previous work that a model informed by mRNA time profiles only could predict experimental TOC1 and CCA1 protein time profiles with great accuracy (Morant et al., 2010). This is because mRNA profiles naturally contain information about the transcription factors modulating their synthesis. In this work, where we focus on the behavior of the clock in LD 12:12 condition, we thus limited the expression time courses used to constrain our mathematical model to microarray expression data for *TOC1*, *CCA1*, *LOV-HK*, and *Rhod-HK*, which were obtained previously under this illumination protocol in white light (Monnier et al., 2010). Doing so guarantees that experimental data are equally available for all four actors. For completeness, we also included a penalty term in the score function used for adjustment to favor correct timings for the protein peaks, however we found that this was a rather weak constraint. Experimental and numerical profiles are compared in Figure 2A. We searched the available genome-wide expression data (Monnier et al., 2010) for transcription factors whose time profiles would match that of the intermediary actor X, and found that some such as the MYB transcription factor fulfill the requirement. We thus included its experimental profile in Figure 2A, however we stress again that identifying X unambiguously is not essential to our analysis, and that a transcription factor with a similar profile would also work. Figure 2A shows an excellent agreement between experimental and numerical time profiles, which provides a first support for the reciprocal regulation of the TOC–CCA1 loop and of the LOV-HK and Rhod-HK putative photoreceptors assumed in our mathematical model. The putative phosphorylation level of TOC1 is shown in the top panel of Figure 2A. It varies little over daytime, even though the concentrations of the two photoreceptors vary significantly. This suggests that the light input pathway operates close to saturation. The predicted protein profiles are shown in Supplementary Figure 2 and are in good agreement with experimental data.

The second dataset used in the adjustment was obtained previously in experiments probing the clock response to resetting (Djouani-Tahri et al., 2010; Troein et al., 2011). In these experiments, cells are entrained under an alternation of white light and darkness before being released into constant light conditions at various phases of the cycle. More precisely, the durations of the light and dark periods are 12 h, except for the last dark period, whose duration varies between 2 and 22 h, similar to an advanced or delayed dawn. The phase of the circadian oscillations is measured 72 h after the end of the last 12-h dark period, by noting the timing of the CCA1 peak relative to that observed when the last dark period lasts exactly 12 h (Supplementary Figure 3). These measurements probe the response of the light input pathway to light during variable amounts of time. Note that the curves so obtained do not necessarily converge to a zero phase shift when the duration of the last night goes to zero. In the 0-h and 12-h last dark period cases, indeed, the clock experiences the same alternation of light and dark before going into constant light, however it spends 24 additional hours in the free-running regime. Therefore, the phase shift measured will be given by the difference between the FRP and 24 h, provided that circadian oscillations have settled to their limit cycle when the phase is measured. The information contained in this resetting curve is very similar to that provided by a phase response curve (PRC) (Johnson et al., 2003), except for the fact that a PRC is relative to a given limit cycle while here, the limit cycle will typically change between the LD 12:12 and constant light conditions. As can be seen in Figure 2B, an excellent agreement between experimental data and the model predictions is also obtained, less so for the last point at 22 h, perhaps because the asymptotic regime has not yet been reached.

Finally, we measured the values of the free-running period under constant light conditions, at different intensity levels in blue or green light (Figure 2C). The time courses recorded in these experiments are shown in Supplementary Figure 4. Constant illumination does not correspond to a physiological situation for the clock, since it was shown that the limit cycle followed by the oscillations depends on photoperiod (Thommen et al., 2012), and thus may differ between light/dark protocols and constant light protocols. Nevertheless, the change of period with light intensity directly reflects the varying influence of the photoreceptors on the central circadian oscillator and thus provides an important piece of information to constrain the model.

Interestingly, the FRP values measured range between 22 and 26 h both in blue light and green light, giving some support to the hypothesis that the signals from the two photoreceptors are integrated before reaching the TOC1-CCA1 oscillator. However, it can be seen that the FRP reacts much more sensitively to blue light than to green light, and saturates more rapidly, with the maximal value of the FRP being reached for intensities as low as 5 μ mol.quanta.m^−2^.s^−1^. This is a first indication that the clock responds differently to blue and green light. These experimental data bring essential information about the sensitivity of the photoreceptors, by characterizing the typical intensity levels at which the clock behavior is affected by input light. Surprinsingly, the mathematical model predicts a non-monotonic variation of the FRP at low light intensity, both in blue and green light, with the FRP values becoming very close to 24 h as light intensity goes to zero. This remarkable result may be related to the dynamical mechanism to shield the clock against daylight fluctuations that we have described in previous works (Thommen et al., 2010, 2012; Pfeuty et al., 2012). This mechanism requires that the limit cycle followed by the clock when is entrained by light/dark cycles changes very little with light intensity. This is more easily achieved when the FRP in the dark is close to 24 h. Another observation hinting at such a mechanism is the fact that the phase shift for a last dark period duration of 0 h is zero. This seems to indicate that the FRP remains close to 24 h for some time after transiting to constant light (as discussed previously, the value at zero is the difference between the FRP and 24 h). This suggests the action of a slow adaptation mechanism tuning the FRP to 24 h under light/dark cycles, avoiding the need for period mismatch compensation in the entrained state, and thus lowering the effet of daylight fluctuations.

An interesting result of the data adjustment procedure is that it provides some information about the spectral response of LOV-HK and Rhod-HK. Table 3 shows the intensities *I*_*L*_ and *I*_*R*_ perceived by the two photoreceptors for 1 μ mol.quanta.m^−2^.s^−1^ of incident light. The coefficients obtained confirm that LOV-HK senses blue light very efficiently (*I*_*L*_ = 1.07). The value *I*_*L*_ = 0.22 in green light seems a bit large given the overlap of the absorption spectrum of LOV-HK and of the emission spectrum of the green LED (Supplementary Figure 1), even though the order of magnitude is correct. This may indicate that the overlap between the LOV-HK expression profile and the TOC1 expression profile is underestimated and needs to be compensated by a higher value of the coefficient. Interestingly, the mathematical analysis predicts that Rhod-HK is a blue-green light receptor, with a slightly higher sensitivity for blue light.

### 2.3. Parameter identification and sensitivity analysis

**Figure 3:**
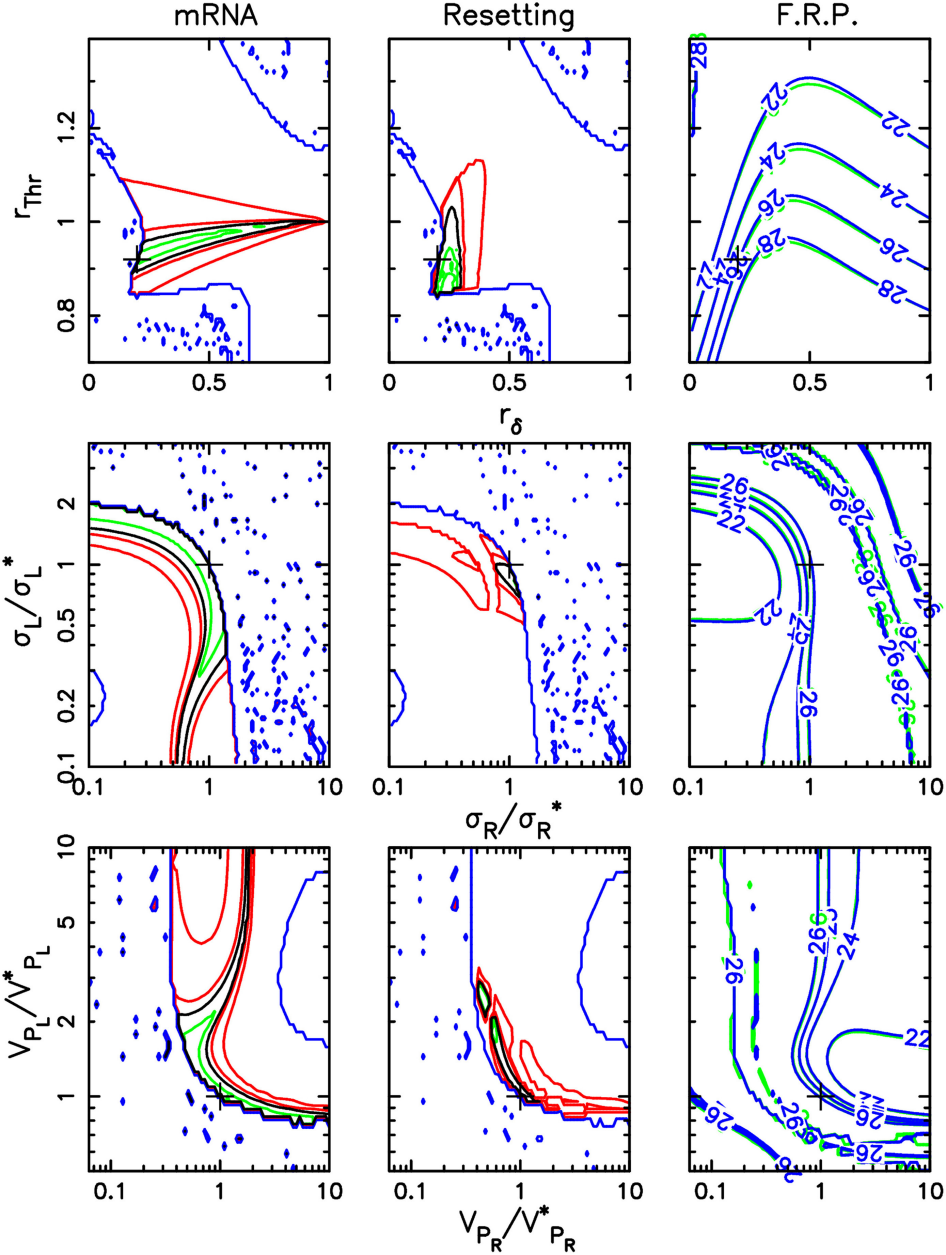
**Sensitivity of adjustment to key control parameters**. This figure recapitulates how the three different datasets constrain the parameters of the mathematical model. For each parameter set, the adjustment procedure computes a score which is the sum of the partial scores for each dataset. Global adjustment thus results from a compromise between the three partial adjustments. The parameter values selected in the global adjustment are indicated by a cross. The first two columns display contour lines of the scores specific to mRNA LD 12:12 time profiles (first column) and to resetting experiments (second column). The third column shows contour lines of the FRP value at high intensity in blue or green light (optimal scores are obtained with FRPs close to 26 h). In each column, the contour lines are displayed in two-parameter planes characterizing how the TCS acts on TOC1 (TOC1 degradation rate and transcriptional activity modulation depths *r*_δ_ and *r*_*Thr*_, first row), sensitivity of the TCS to light input in blue and green (σ_*R*_/σ^*^_R_ and σ_*L*_/σ^*^_L_, where σ^*^_R_ and σ^*^_L_ are the best-fit sensitivities, second row) and photoreceptor activation window (Rhod-HK and LOV-HK maximum degradation rates *V*_*P*_*R*__/V^*^_P_*R*__ and *V*_*P*_*L*__/V^*^_P_*L*__, where V^*^_P_*R*__ and V^*^_P_*L*__ are the best-fit maximum degradation rates, third row). In the first and second columns, the black line indicates the level line corresponding to the partial score for that dataset that was obtained for the best fitting parameter set in the global adjustment, and which serves as a reference. Green contour lines correspond to partial scores equal to 95, 90, and 85% of this reference score (when applicable), and delimitate regions where adjustment would be improved if only this dataset were considered. Red contour lines correspond to partial scores equal to 110 and 150% of the reference score and provide information about how quickly the partial scores degrade away from the optimum.

Since we use different types of data in the adjustment procedure, it is interesting to assess how they constrain the mathematical model and the values of the control parameters, allowing us to identify the most informative measurements. In Figure 3, we show how the individual goodnesses of fit for time profiles, resetting, and FRP vary depending on selected control parameters. These parameters comprise the variation of TOC1 half-life and transcriptional activity in response to its phosphorylation level, which characterize the action of the light input pathway on the core oscillator, as well as the sensitivities and the half-lifes of the two photoreceptors, which characterize the strength and timing of the light signal reaching the oscillator. In the plots of the left and center column, we indicate where the global best fitting parameter set is located, as well as how the adjustment error could be lowered by considering only the corresponding dataset. This allows one to estimate how different parameters are constrained by the different datasets. Remarkably, general remarks can be made independently of the parameters considered.

First, adjustment of the model to the experimental time profiles only brings partial information (Figure 3, first column). For parameters characterizing photoreceptors, optimal adjustment is found over wide regions of parameter space. However, typically, one of the parameters is essentially fixed once a value is picked for the other one. In particular, this may indicate that, for example, modulations of TOC1 stability or transcriptional activity have similar dynamical effects. In contrast with this, the resetting experiments (Figure 3, second column) are seen to constrain the model parameters much more and restrict them to a relatively small region of parameter space. The free-running period measurements are similar to the time profile data in that they only force the relative values of the two parameters considered but not their absolute values. Interestingly, the three datasets constrain the control parameters differently, but consistently so that an excellent global adjustment can be obtained.

### 2.4. Model predictions for FRP and entrainment phase

**Figure 4:**
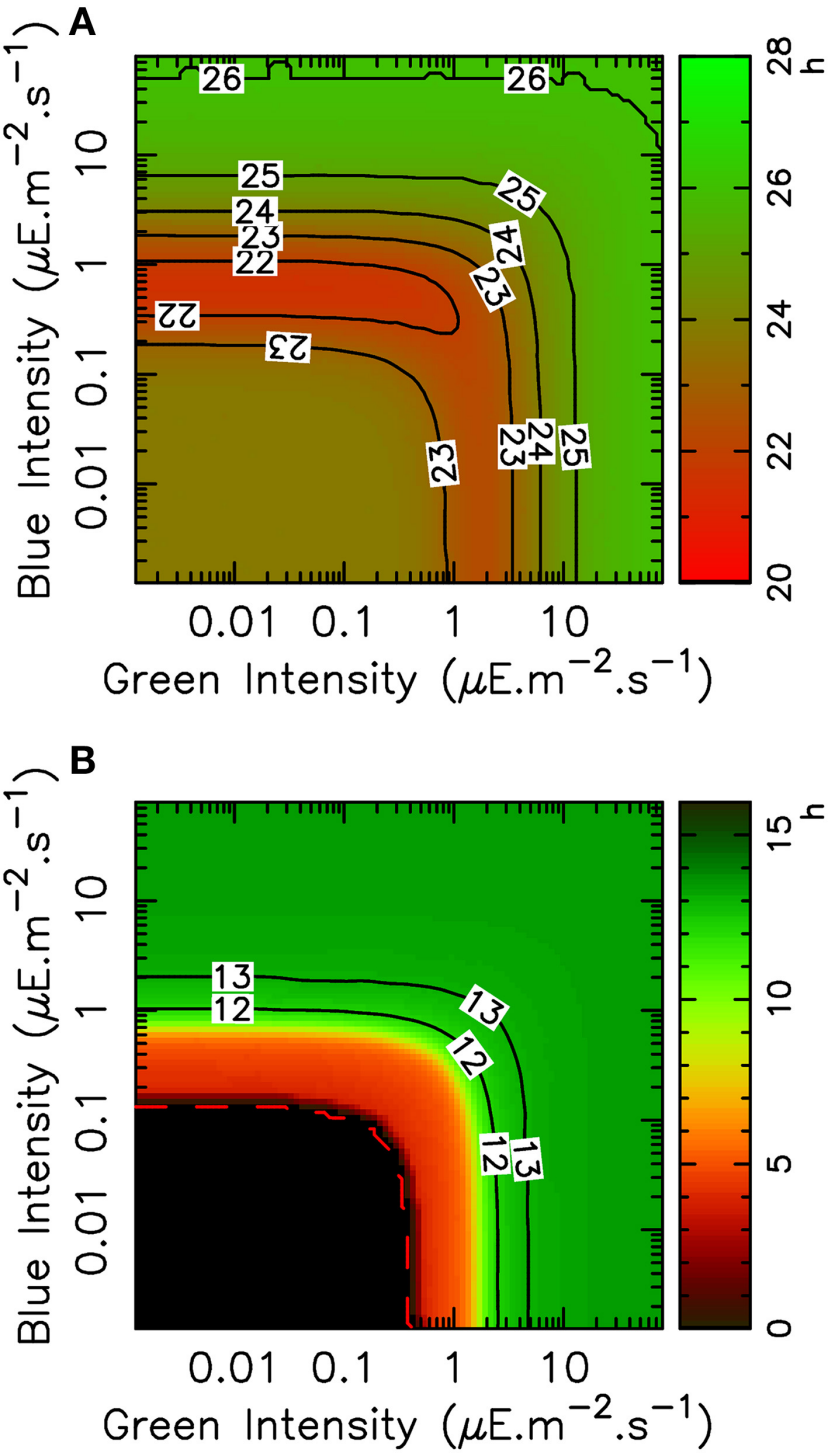
**Variation of FRP and TOC1 expression peak timing with blue and green light intensities in the mathematical model**. **(A)** Variation of the free-running period with light intensities in green and blue light, shown as a heat map and with level lines (solid black lines). **(B)** Timing (ZT) of the TOC1 expression peak in LD 12:12 entrainment condition as a function of light intensities in green and blue light, shown as a heat map with level lines (solid black). The black area indicates the region where the circadian clock does not lock to the light/dark cycle.

Experiments are usually carried out with only a few different light qualities, typically either white, blue or green light. In particular, our mathematical model was only informed by FRP values in pure blue or pure green light. In the sea, however, *O. tauri* is typically subjected to continuously varying proportions of blue and green light as it changes its distance to surface, due to differential absorption of light by water. It is therefore interesting to use our mathematical model to predict how the clock reacts to arbitrary combinations of blue and green light. Figure 4 shows how the free-running period (top panel) and timing of the TOC1 protein peak under entrainment (bottom panel) depend on blue and green light intensity in numerical simulations.

Consistently with Figures 2C, 4A shows that the FRP is more sensitive to blue light than to green light, in the sense that less of blue light is required to reach a given FRP value. Moreover, blue and green sensing are relatively independent, in that FRP depends essentially on only one intensity until the other one is sufficiently high, as indicated by the fact that level lines are globally either vertical or horizontal (regardless of the non-monotonicity observed in some regions of the diagram). For example, a FRP of 24 h is obtained with approximately 3 μ mol.quanta.m^−2^.s^−1^ of pure blue light. If green light is then added, the FRP will almost not change until the green light intensity reaches around 6 μ mol.quanta.m^−2^.s^−1^, and then will increase depending on the green light intensity only. Globally, one color or the other is dominant in determining the FRP.

Similar observations can be made for the TOC1 protein peak timing as a function of blue and green intensities (Figure 4B), also with a higher sensitivity to blue light. Phase locking is predicted to occur for intensities of 0.1 μ mol.quanta.m^−2^.s^−1^ in blue light and 0.4 μ mol.quanta.m^−2^.s^−1^ in green light. The physiological TOC1 peak timing at zeitgeber time (ZT) 13 is obtained for intensities of 2 μ mol.quanta.m^−2^.s^−1^ and higher in blue light but requires around 5 μ mol.quanta.m^−2^.s^−1^ in green light.

**Figure 5:**
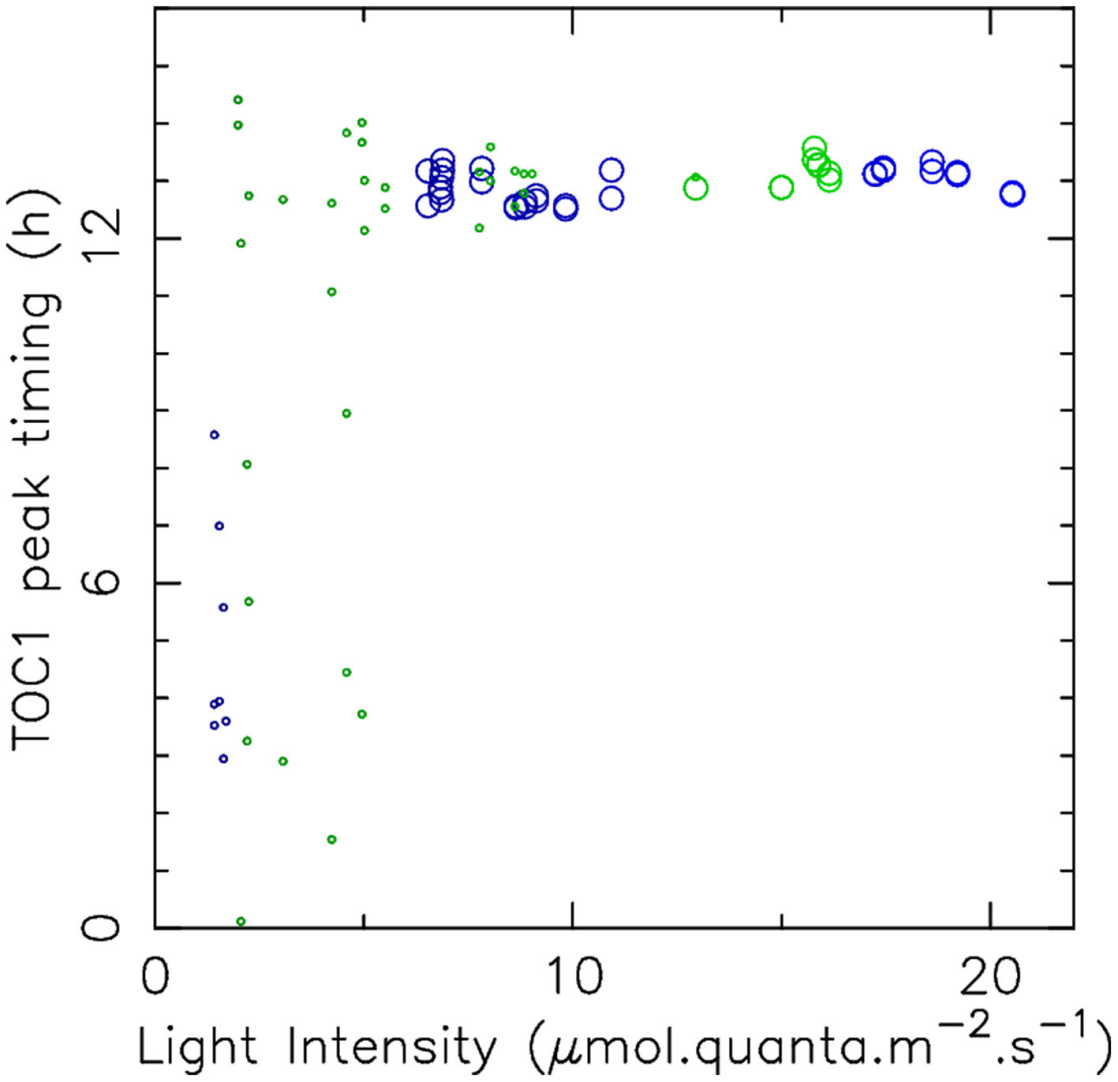
**Dependance of entrainment on light intensity**. The timing of TOC1 expression peak under LD 12:12 cycles is displayed as a function of light intensity for green light (green circles) and blue light (blue circles). Large circles correspond to situations where large amplitude, reproducible, oscillations indicate stable entrainment. Small circles indicate cases where the luminescence signal was low, with no oscillations clearly discernible. Raw data are displayed in Supplementary Figure 5.

These theoretical predictions were tested experimentally by subjecting *O. tauri* cell cultures to an alternation of light and dark phases of 12 h, under various intensities of blue and green light clustered around 2, 7, and 15 μ mol.quanta.m^−2^.s^−1^. The raw time courses are shown in Supplementary Figure 5. In both green and blue light, we found that luminescence signals remain close to the background noise for lower illumination intensities (Supplementary Figure 5). This may indicate that in those conditions, the energy content of light is not sufficient to sustain the transcriptional machinery. For higher intensities, stable entrainment is observed with a TOC1 peak timing which almost immediately saturates to ZT13, as can be seen in Figure 5, which displays TOC1 peak timing as a function of light intensity. This allows us to estimate the physiological entrainment threshold to be between 2 and 7 μ mol.quanta.m^−2^.s^−1^ in blue light and between 7 and 12 μ mol.quanta.m^−2^.s^−1^ in green light. Keeping in mind that the metabolic role of light may here interfere with its signaling role, this is reasonably close to the theoretical estimates and is sufficient to assess the environmental conditions under which entrainment can be achieved. A higher sensitivity to blue light is also observed here, essentially in the same proportion as in numerical simulations.

### 2.5. Resetting in blue and green light : theory and experiments

To understand how *Ostreococcus* clock can keep time in different light qualities, it is important to study how it can be reset by blue and green light. We therefore determined the phase shift experienced by the clock when dawn is advanced or delayed, under blue and green light, experimentally and numerically. To take into account the different sensitivities to blue and green light, the intensities used were fixed at 10 μ mol.quanta.m^−2^.s^−1^ in blue light and 25 μ mol.quanta.m^−2^.s^−1^ in green light. The protocol followed was the same as for the resetting experiments in white light described in Section 2.2 (Figure 2B and Supplementary Figure 3).

**Figure 6:**
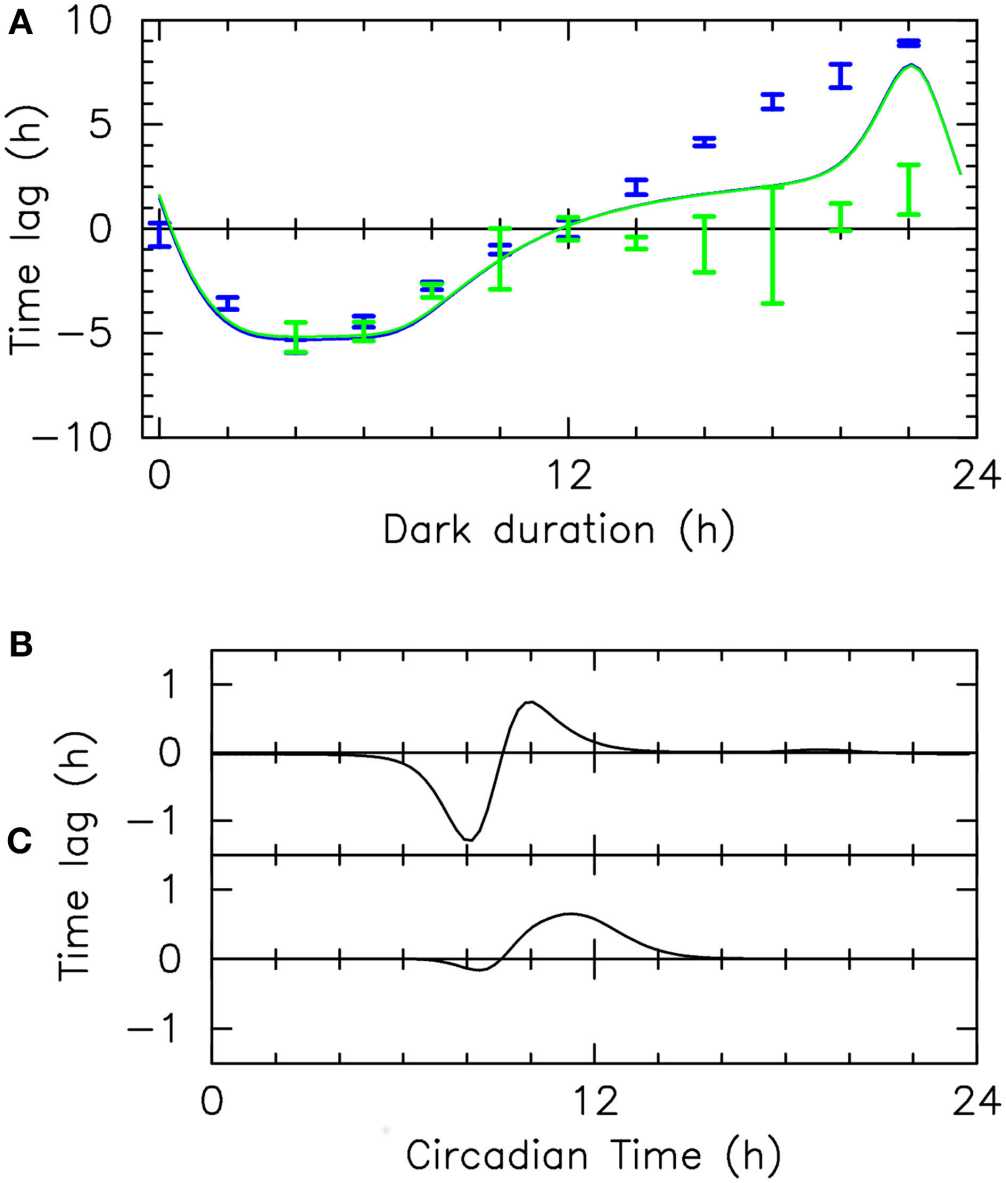
**Resetting: numerical simulations and experiments**. **(A)** Phase shifts measured in resetting experiments in blue and green light (blue and green vertical bars, respectively) and corresponding numerical estimates (blue and green solid lines, respectively), as a function of the duration of the last dark period. This phase shift is the difference of the asymptotic phase with that obtained for a 12-h last dark period. Blue (resp., green) light intensity is 10 (resp., 25) μ mol.quanta.m^−2^.s^−1^ **(B–C)** Phase response curves giving the phase shift observed in numerical simulations for an intense 1-h light pulse when only LOV-HK **(B)** or Rhod-HK **(C)** is active. These phase response curves characterize the role of the two photoreceptors in resetting the clock.

Figure 6A shows the experimental and numerical phase shift observed as a function of the last dark period duration. When dawn is advanced (last dark duration between 0 and 12), the agreement between experimental and numerical values is excellent. An important observation is that the experimental phase shifts obtained in blue, green or white light (Figures 2, 6A) are identical. Independently of mathematical modeling, this fact supports the TCS hypothesis since it indicates that the input pathway integrates input from all photoceptors and that it is saturated at the intensities used, as would typically be the case for a TCS. However, note that there is a small discrepancy when the last dark period duration is zero (i.e., when the last light/dark cycle is skipped). Contrary to the numerical estimates, the experimental phase shifts go to zero, as with white light in Figure 2B. As discussed above, this may be the signature of a strategy to make the clock immune to daylight fluctuations (Thommen et al., 2010, 2012; Pfeuty et al., 2012).

When dawn is delayed (duration of last dark period larger than 12 h), however, our mathematical model poorly predicts the resetting experienced by the clock. Numerical estimates in green and blue light remain identical, and are also close to the values predicted for white light, suggesting a saturated response. However, it can be seen that the experimental responses in the two light qualities are very different, with a much higher sensitivity to blue light. In green light, the experimental phase shift remains very small except when the last dark duration approaches 22 h, and even then remains modest. In blue light, there is a strong resetting, with a maximal phase shift of about 9 h. Interestingly, this value is significantly higher than in white light (Figure 2B). The theoretical phase shift is half-way between the blue-light and green-light phase shifts but comes closer to the blue-light phase shift when the last dark period duration approaches 22 h. This suggests that in experiments, the input pathway is not saturated, in contrast with numerical simulations. This is consistent with the fact that during the subjective day, which is the time period probed by delayed dawn experiments, TOC1 levels are very low. Moreover, the overlap of TOC1 levels with LOV-HK levels is presumably higher than with Rhod-HK levels during this period, which may qualitatively explain the different in resetting between blue and green light.

Since we found that the two photoceptors sense both blue and green light, although with different sensitivities, it was interesting to characterize how each photoreceptor contributes to resetting. To this aim, we computed the response of our mathematical clock model to strong 1-h light pulses saturating the input pathway, assuming that one or the other photoreceptor is inactivated, so as to mimick the effect of a receptor knock-out. Figure 6B (resp. Figure 6C) shows the phase response curve obtained when only LOV-HK (resp., Rhod-HK) is active, which describes whether the 1-h pulse elicits a phase advance or delay. According to the mathematical analysis, both receptors contribute to resetting, however they do it in different ways. LOV-HK is more effective in advancing the phase (mostly around CT8), while Rhod-HK can only delay the phase (and thus would only be able to entrain a fast-running clock). This allows one to understand the non-monotonic behavior of the FRP with light intensity. At low intensities, LOV-HK is more sensitive and thus the FRP decreases with intensity. As light intensity increases and LOV-HK saturates, the influence of Rhod-HK is more and more important, leading then to an increase in the FRP. It would be interesting to study whether these contrasting actions of the LOV-HK and Rhod-HK photoreceptors, together with their slightly different spectral response (Rhod-HK absorption spectrum appears to be more evenly spread between the blue and green frequency ranges), confer some type of robustness to the clock.

## 3. Discussion

Most organisms live in an environment which changes periodically with a period of 24 h. The vast majority of them rely on a circadian clock to keep track of time and schedule various processes appropriately. Daylight is the major environmental cue for most circadian clocks, at least at organismal level. It is indeed a good indicator of day/night status, although it displays day-to-day fluctuations due to weather as well as variations all along the year, which the circadian clock must accommodate. Therefore, circadian clock molecular networks typically incorporate photoreceptor proteins, which change their state and activity in response to light.

However, there is more in circadian photoreception than just capturing day/night status, which would require only one photoreceptor. Indeed, most circadian clocks feature several photoreceptors, of different types (Panda et al., 2003). Moreover, these photoreceptors often operate in different spectral ranges (as is, e.g., the case for the phytochromes and cryptochromes of Arabidopsis), which suggests that light carries different information at different wavelengths, and that being able to resolve light spectrally is beneficial to circadian clocks and biological time-keeping (Falciatore and Bowler, 2005). Light quality is especially significant for marine organisms since the light spectrum they perceive typically depends on distance to sea surface and water turbidity. In the open sea, light is less absorbed in the blue, which becomes thus predominant as depth increases. In coastal waters, the phytoplanktonic chlorophyll pigments, as well as the so-called “yellow substances” produced by phytoplankton metabolism, induce absorption in the blue and red part of the spectrum, so that green is the dominant color (Pelevin and Rutkovskaya, 1977; Prieur and Sathiendranath, 1981; Morel, 1988). It is thus tempting to hypothesize that the combined information provided by several photoreceptors operating in different spectral regions may help the circadian clock to function in very different light conditions.

*O. tauri* tauri is a particularly interesting organism to study these questions. *O. tauri* is a photosynthetic marine organism, implying that light and its daily rhythms play a central role in its metabolism, and that it is potentially subjected to large fluctuations in daylight quality. Previous studies have identified a central circadian oscillator based on orthologs of *Arabidopsis* TOC1 and CCA1 clock genes, suggesting that *O. tauri* clock may be a reduced version of *Arabidopsis* clock. Moreover, several light-sensitive proteins have been identified in *O. tauri* and are thus putative photoreceptors. Of particular interest are the two histidine kinases LOV-HK and Rhod-HK, which have been hypothesized to serve as sensors of a two-component signaling cascade where a phosphorelay protein (HPT) would integrate signals from these two kinases to modify TOC1 post-translationally. LOV-HK is a blue light receptor under circadian control, whose functional role in the circadian clock has been demonstrated previously (Djouani-Tahri et al., 2011). Rhod-HK, besides of being a putative upstream actor of TOC1, features a rhodopsin domain which is plausibly sensitive in the blue-green domain, as in the fruit fly (Salcedo et al., 1999). Finally, previous modeling studies have shown that *O. tauri* was more amenable to quantitative modeling, probably because of its relatively simple cellular organization and its low genomic redundancy (Morant et al., 2010; Troein et al., 2011; Thommen et al., 2012).

In this work, we used mathematical modeling to test the hypothesis of a two-component signaling cascade based on LOV-HK and Rhod-HK transmitting light information to the TOC1-CCA1 oscillator via TOC1 post-translational modifications. More precisely, we have shown that a minimal model incorporating this TCS with the TOC-CCA1 loop can adjust well various experimental data of different types, not only mRNA time profiles in LD 12:12 experiments, but also phase shifts from resetting experiments in white light and FRP values measured in different constant intensities of blue and green light. To construct this model, we hypothesized simple regulations of the two photoreceptors by the TOC-CCA1 loop, which are compatible with the observed LOV-HK and Rhod-HK mRNA profiles. This shows that circadian control of the two light sensors by the core oscillator is plausible, but the assumed interactions are speculative at this stage and should be further tested. We also showed that time profiles, resetting experiments and FRP measurements bring complementary information, and that the latter constrain the mathematical model very much, as FRP are sensitive to control parameters.

An interesting result of data adjustment is that Rhod-HK seems to be a blue light photoreceptor with also some sensitivity in the green spectral range (Table 3). Remarkably, this spectral response appears to be very close to that of the blue-sensing state of the *Chlamydomonas* histidine-kinase rhodopsin receptor (Luck et al., 2012). At this stage, however, it should be kept in mind that an accurate determination of these coefficients depends on how the protein time profiles are well reproduced and also on how faithful the description of the response of TOC1 phosphorylation to light is. For example, the relatively high sensitivity to green light found for LOV-HK contrasts with the small overlap of the absorption spectrum of LOV-HK with the green LED emission spectrum. This may result from an underestimation of LOV-HK protein levels near the end of the day.

An important prediction of the mathematical model is that both photoreceptors work in a relatively independent way even though their signals are integrated before reaching TOC1. By computing numerically how the FRP or the entrained TOC1 peak timing vary as a function of blue and green intensities, we found that typically, the intensity in one quality or in the other determines these properties alone. Whether it is blue or green light that is driving the clock depends on their relative intensities. Another interesting prediction is that the clock is generally more sensitive to blue light than to green light, in that much smaller amounts of blue light are needed to achieve physiological TOC1 peak timing or the maximal FRP value. This is generally consistent with experiments, except in resetting experiments when dawn is advanced, where the response to blue and green light is similar due to pathway saturation. Altogether, this suggests that *O. tauri* clock has evolved to operate in different light conditions, where either blue light (open sea) or green light (coastal waters) is dominant, but with little interference between the two signals. The difference in sensitivity between blue and green light may be linked to the fact that the latter typically dominates in shallow waters, where light intensity is much higher.

How *O. tauri* clock simultaneously discriminates and integrates light spectral information to control TOC1 regulatory activity and stability is reminiscent of the light input pathway of an other green alga, *Chlamydomonas*. In this organism, the transcription factor ROC15 is not only an essential component of the clock (Matsuo et al., 2008) but is also the main trigger of phase resetting through its light-induced phosphorylation and subsequent degradation (Niwa et al., 2013). This resetting mechanism operates over broad spectrum of wavelength, suggesting convergent light signaling mediated by multiple photoreceptors. Noteworthily, the resetting efficacy as a function of the wavelength of light seems to differ depending on the strain or on adaptation to prior lightning conditions (Johnson et al., 1991; Kondo et al., 1991; Niwa et al., 2013; Forbes-Stovall et al., 2014), raising the question of the adaptive capabilities of the wavelength-dependent resetting properties in *O. tauri* as well.

To validate our mathematical model, we predicted the response of the clock when it is switched from LD 12:12 cycles to blue or green constant light, and measured this response experimentally. This is the first time that the influence of light quality on the resetting capacity of *O. tauri* clock is characterized biologically. We found an overall good match between experimental and theoretical phase shift curves. The agreement is excellent for advanced dawn experiments, both in blue and green light. This suggests that important features of the light input pathway have been captured. In particular, the fact that the same phase shifts are observed in white, blue or green light suggests that all light qualities are integrated in a unique pathway which saturates at high intensity, exactly as predicted by the TCS hypothesis independently of mathematical modeling.

In delayed dawn experiments, however, the mathematical model strongly underestimates phase shifts measured in blue light and overestimates those measured in green light. This suggests that in these experiments, which probe the expression time profiles during the subjective day, during which TOC1 levels are typically low, the input pathway is far from being saturated and that the overall higher sensitivity to blue light prevails. The discrepancy observed might thus stem from an inaccurate prediction of the photoreceptor and TOC1 protein time profiles, especially as little is known about Rhod-HK. Indeed, those protein levels critically control the amount of resetting, unless the signaling cascade is saturated. A puzzling fact is that blue light resets the clock significantly more than white light when the last dark period lasts almost 24 h, which may be due to the presence of another photoreceptor not yet identified.

Yet, the overall good agreement between experiments and numerical simulations all the more supports the TCS hypothesis that there are known limitations in the structure of our mathematical model. To keep it as simple as possible in this first assessment of the TCS hypothesis, we indeed did not try to endow it with the dynamical mechanisms which were shown to provide robustness to daylight fluctuations (Thommen et al., 2010, 2012). Moreover, it was shown that the limit cycle followed by oscillations depends on photoperiod (Thommen et al., 2012) independently of the fast light inputs, and thus that it may change significantly between LD 12:12 and LL conditions, which is not taken into account in our model. There are some indications in our experimental data that such mechanisms are indeed active. In particular, the phase difference between resetting experiments where the duration of the constant light phase differs by 24 h is significantly smaller than expected. This indicates that the FRP remains close to 24 h for some time after release into constant light, suggesting that there is an adaptation mechanism which tunes the FRP to 24 h when the clock is entrained by light/dark cycles. Also, the mathematical model predicts that the FRP becomes very close to 24 h at zero light intensity (which cannot be tested experimentally since transcription is inhibited in the dark). Such mechanisms would contribute to making the clock robust to daylight fluctuations even if they are not strictly necessary (Thommen et al., 2010). In our future works, we intend to incorporate such mechanisms in our mathematical model. To improve the prediction of delayed dawn experiments, in which the light input pathway is not saturated, it would also be important to better describe the TOC1 and photoreceptor protein time profiles, as well as how they overlap. In particular, the fact that blue light can induce LOV-HK expression (Djouani-Tahri et al., 2011) should be taken into account. Another interesting extension of the present work would be to carry the same study for different photoperiods, as we showed previously that the TOC1 and CCA1 expression profiles vary significantly with the photoperiod (Thommen et al., 2012).

To conclude, a minimal mathematical model incorporating a putative light input pathway based on the TCS hypothesis reproduces relatively well mRNA expression profiles, response to resetting experiments and FRP values in different light qualities. This provides support for the hypothesis that a two-component signaling cascade based on the light sensors LOV-HK and Rhod-HK and modifying TOC1 post-translationally plays an important role in synchronizing *O. tauri* clock to the day/night cycle. Our results thus confirm that *O. tauri* is a very promising model organism for systems biology studies of environmental issues in marine organisms.

## 4. Materials and methods

### 4.1. Experiments

*O. tauri* CCA1-Luc and TOC1-Luc translational reporter lines have been previously described (Corellou et al., 2009; Djouani-Tahri et al., 2011). Cultures were grown to saturation in microplate under LD conditions or constant light. Cells were plated at equal cell density (10.10^6^ cell/ml) in the culture medium containing D-luciferin as previously described (Corellou et al., 2009). Cell synchronization was achieved by at least 3 12:12 LD cycles before placing the cells under either blue light LEDs (462 nm maximum) or green light LEDs (521 nm maximum). Luminescence was acquired for 5 s every hour using an automated microplate luminometer (Berthold LB Centro). Resetting experiments were performed by transferring cells to constant darkness or light at various time of the day/night cycle (Troein et al., 2011).

### 4.2. Mathematical model

The mathematical model describing the dynamics of the molecular network shown in Figure 1 consists of the following ten differential equations:

(1a) $$\dot{M_{T}}=\mu_{T}+\frac{\lambda_{T}}{1+{\left(P_{C}/T_{T}\right)}^{2}}-V_{M_{T}}\frac{M_{T}}{K_{M_{T}}+M_{T}}$$

(1b) $$\dot{P_{T}}=\beta_{T}M_{T}-\left(1-\alpha +r_{\delta }\alpha \right)V_{P_{T}}\frac{P_{T}}{K_{P_{T}}+P_{T}}$$

(1c) $$\begin{array}{l} \dot{M_{C}}=\mu_{C}+\frac{\lambda_{C}{\left(\left(1-\alpha +r_{Thr}\alpha \right)P_{T}/T_{C}\right)}^{2}}{1+{\left(\left(1-\alpha +r_{Thr}\alpha \right)P_{T}/T_{C}\right)}^{2}}\\ \;-V_{M_{C}}\frac{M_{C}}{K_{M_{C}}+M_{C}} \end{array}$$

(1d) $$\dot{P_{C}}=\beta_{C}M_{C}-V_{P_{C}}\frac{P_{C}}{K_{P_{C}}+P_{C}}$$

(1e) $$\dot{M_{R}}=\mu_{R}+\frac{\lambda_{R}}{1+{\left(P_{C}/T_{R}\right)}^{2}}-V_{M_{R}}\frac{M_{R}}{K_{M_{R}}+M_{R}}$$

(1f) $$\dot{P_{R}}=\beta_{R}M_{R}-V_{P_{R}}\frac{P_{R}}{K_{P_{R}}+P_{R}}$$

(1g) $$\dot{M_{X}}=\mu_{X}+\frac{\lambda_{X}}{1+{\left(P_{C}/T_{X}\right)}^{2}}-V_{M_{X}}\frac{M_{X}}{K_{M_{X}}+M_{X}}$$

(1h) $$\dot{P_{X}}=\beta_{X}M_{X}-V_{P_{X}}\frac{P_{X}}{K_{P_{X}}+P_{X}}$$

(1i) $$\dot{M_{L}}=\mu_{L}+\frac{\lambda_{L}}{1+{\left(P_{L}/T_{L}\right)}^{2}}-V_{M_{L}}\frac{M_{L}}{K_{M_{L}}+M_{L}}$$

(1j) $$\dot{P_{L}}=\beta_{L}M_{L}-V_{P_{L}}\frac{P_{L}}{K_{P_{L}}+P_{L}}$$

where post-translational modifications are considered to be slaved to kinase activity level and to light input, assuming that the dynamics of the phosphate transfer is fast compared to that of the transcriptional-translational loops. They are taken into account through the parameter α, described later, which denotes the fraction of TOC1 which is phosphorylated.

Equation (1) describe the time evolution of mRNA concentrations *M*_*T*_, *M*_*C*_, *M*_*R*_, *M*_*X*_, and *M*_*L*_ and protein concentrations *P*_*T*_, *P*_*C*_, *P*_*R*_, *P*_*X*_, and *P*_*L*_ for the *Toc1*, *Cca1*, *RodHK*, *X*, and *LovHK* genes, as it results from regulated mRNA synthesis, translation and enzymatic degradation. For each species *Y*, the translation occurs at rate β_*y*_; the transcription rate varies between μ_*Y*_ and μ_*Y*_ + λ_*Y*_ according to the usual gene regulation function with a cooperativity of 2 and a threshold *T*_*Y*_ ; the *V*_*M*_*Y*__ and *K*_*M*_*Y*__ define the Michaelis-Menten degradation term for mRNA; the *V*_*P*_*Y*__ and *K*_*P*_*Y*__ define the Michaelis-Menten degradation term for proteins.

The fraction α of phosphorylated TOC1 depends on the intensities *I*_*R*_ and *I*_*L*_ perceived by Rhod-HK and LOV-HK, and on the expression levels of the two kinases. It is given by:

(2) $$\alpha =\frac{{\left(\sigma_{R}P_{R}\frac{I_{R}}{I_{R_{0}}+I_{R}}\;+\;\sigma_{L}P_{L}\frac{I_{L}}{I_{L_{0}}+I_{L}}\right)}^{4}}{1+{\left(\sigma_{R}P_{R}\frac{I_{R}}{I_{R_{0}}+I_{R}}\;+\;\sigma_{L}P_{L}\frac{I_{L}}{I_{L_{0}}+I_{L}}\right)}^{4}},$$

where *I*_*R*_0__ (resp., *I*_*L*_0__) denote the Rhod-HK (resp., LOV-HK) saturation intensity, $\frac{I_{R}}{I_{R_{0}}+I_{R}}$ (resp. $\frac{I_{L}}{I_{L_{0}}+I_{L}}$) being the fraction of phosphorylated Rhod-HK (resp. LOV-HK). The intensities *I*_*R*_ and *I*_*L*_ are determined from the intensity of the light source using the coefficients whose values, determined from the adjustment, are given in Table 3. For example, when cells are illuminated by 1 μ mol.quanta.m^−2^.s^−1^, *I*_*L*_ = 0.22 and *I*_*R*_ = 0.21. The parameters σ_*R*_ and σ_*L*_ characterize the efficiency of the phosphate group transfer from the two kinases to TOC1.

### 4.3. Model adjustment

Model adjustment was carried out by picking at random a large number of parameter sets in the 41-dimensional parameter space, and using them as starting points for an optimization procedure based on a modified Levenberg–Marquardt algorithm (routine LMDIF of the MINPACK software suite Moré et al., 1999). Goodness of fit for a given parameter set was estimated with the root mean square (RMS) error between experimental and numerical mRNA levels, phase shift values in resetting experiments, and FRP values in green or blue light of various intensities. Numerical integration was performed with the SEULEX algorithm (Hairer and Wanner, 1996).

Because we had no information about absolute expression levels, two parameter sets of Equation (1) which yield the same time profiles up to a scale factor are considered equivalent. The parameter values reported in Tables 1, 2 were fixed so that so that the maximum values of mRNA and protein profiles are 100 and 10 nM, respectively.

## Author contributions

QT, BP, FYB, and ML designed the research. PS, AB, and FYB carried out the biological experiments. QT carried out the numerical experiments. QT, BP, FYB, and ML analyzed the data. QT, BP, FYB, ML wrote the paper.

### Conflict of interest statement

The authors declare that the research was conducted in the absence of any commercial or financial relationships that could be construed as a potential conflict of interest.
