# Supplementary material for: Probing entrainment of Ostreococcus tauri circadian clock by green and blue light through a mathematical modeling approach
Source: Front Genet. 2015 Feb 27;6:65. doi: 10.3389/fgene.2015.00065 (PMC4343026; doi:10.3389/fgene.2015.00065)
Supplement: Supplementary file 1 [file DataSheet1.PDF]

Frontiers in Genetics

Original Research
5 February 2015

Supplementary Material: Probing entrainment of *Ostreococcus tauri* circadian clock by green and blue light through a mathematical modeling approach

Quentin Thommen [1], Benjamin Pfeuty [1], Philippe Schatt [2], Amandine Bijoux [2], François-Yves Bouget [2], and Marc Lefranc [1,*]

[1] *Laboratoire de Physique des Lasers, Atomes, et Molécules, Unité de Formation et de Recherche en Physique, Université Lille 1 Sciences et Technologies, Villeneuve d'Ascq, France*
[2] *LOMIC, Laboratoire d'Océanographie Microbienne, Observatoire Océanologique de Banyuls, Université Pierre et Marie Curie (Paris 06), Sorbonne Universités, Centre National de la Recherche Scientifique, Unité Mixte de Recherche 7621, Banyuls sur Mer, France*

Correspondence*:
Marc Lefranc
Laboratoire PhLAM, Bâtiment P5, Université Lille 1, F-59655 Villeneuve d'Ascq, France, marc.lefranc@univ-lille1.fr

1 SUPPLEMENTARY FIGURES

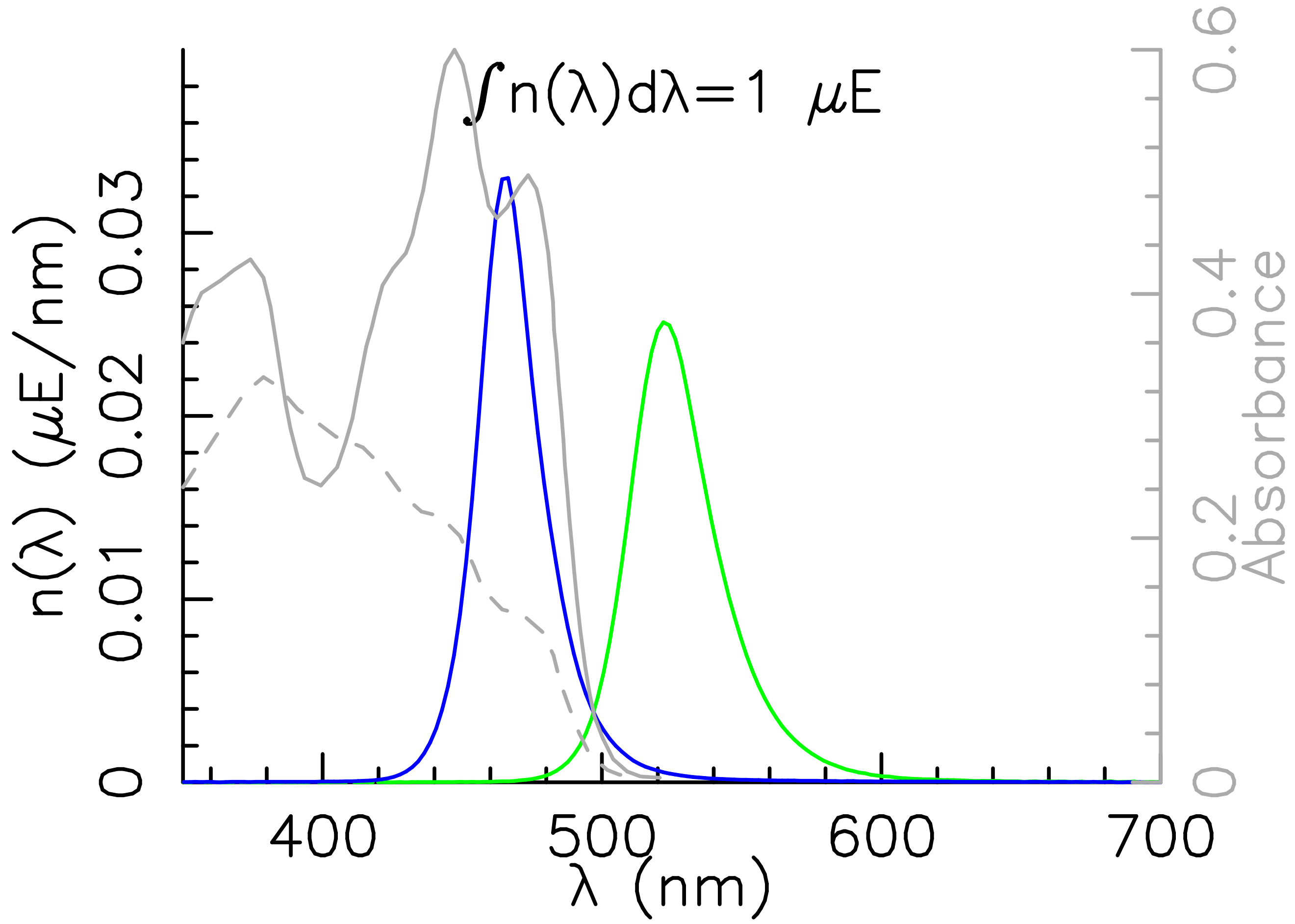

Supplementary Figure 1. Blue (resp. green) solid lines: Emission spectrum of the blue (resp. green) LED. Absorption spectrum of the *O. tauri* LOV domain fused to a glutathione sepharose tag in the dark (full line) and after a light pulse (dashed line) (adapted from Ref. (1)).

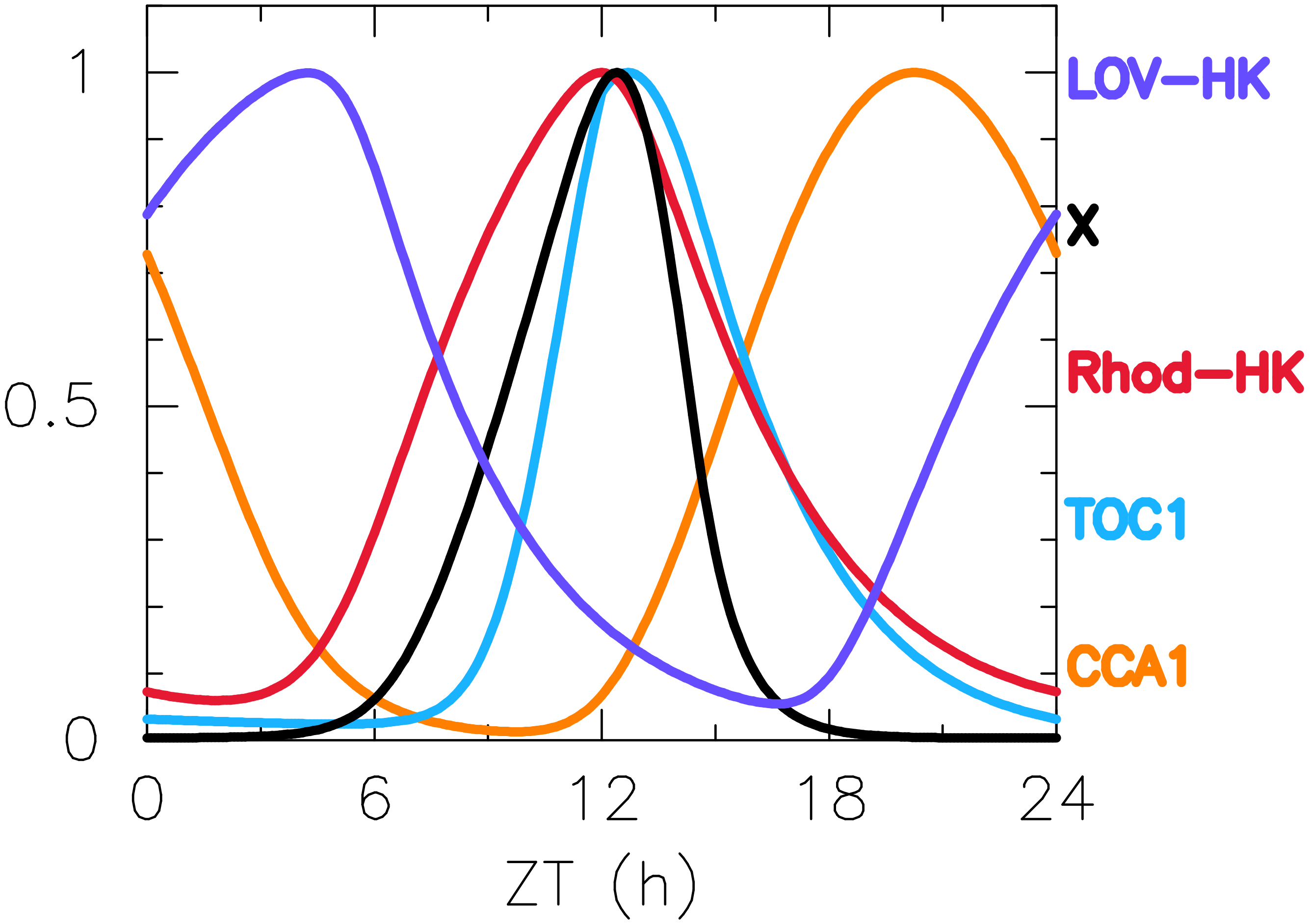

Supplementary Figure 2. Protein time profiles for the molecular actors of the network of Fig. 1 as predicted by the mathematical model in LD 12:12 cycles with intensities in blue and green light of 17.5 $\mu Em^{-2}s^{-1}$. Profiles are normalized to their maximum value.

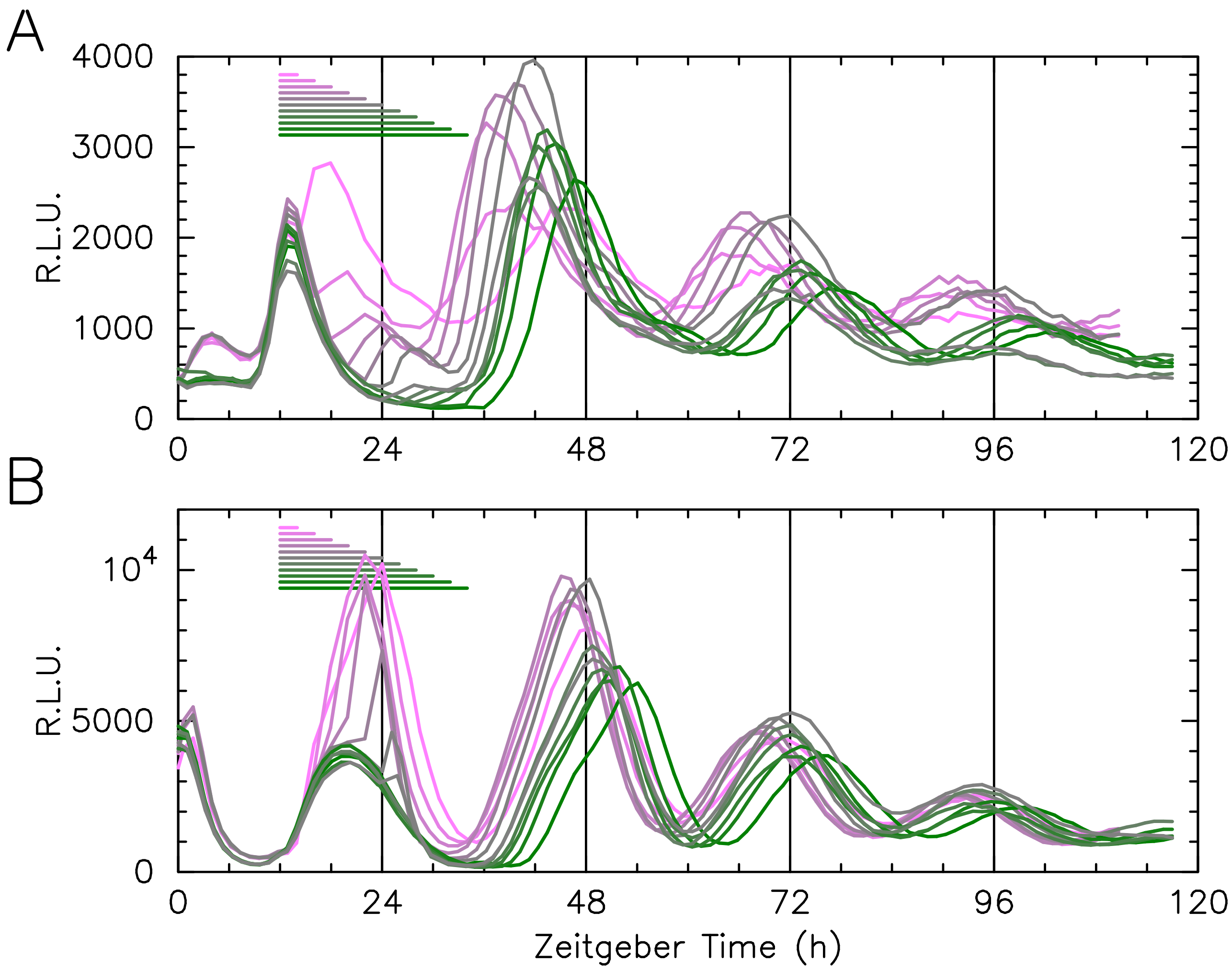

Supplementary Figure 3. Experimental data from resetting experiments in white light Cells are synchronized using LD 12:12 cycles (light intensity: 35 μmol.quanta.m^{-2}.s^{-1}, approximately equally distributed between blue and green), then are exposed at time 0 to a light period of 12 hours, a dark period of variable length and released into constant light conditions with an intensity of 15 μmol.quanta.m^{-2}.s^{-1}. The circadian oscillations are monitored using (A) TOC1:luc luminescent reporter; (B) CCA1:luc luminescent reporter. The last dark time interval is indicated by a bar in the inset, and is color coded according to duration. The same color code is used for the time traces. The phase of oscillations is measured at time 72 and is reported relative to the phase obtained when the last dark period lasts 12 hours. Resetting experiments in blue or green light are carried out following the same protocol.

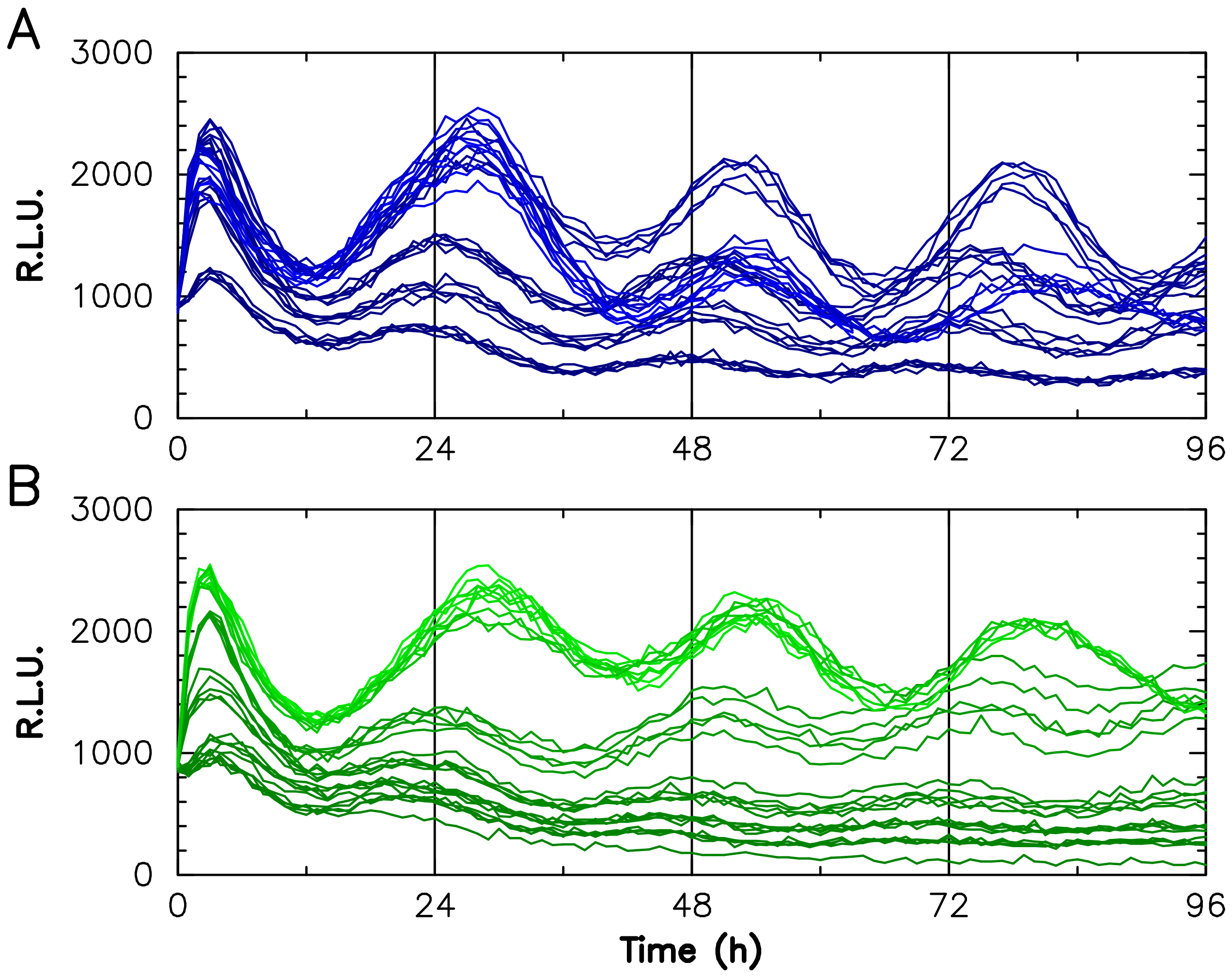

Supplementary Figure 4. Experimental time courses for FRP measurements. CCA1:luc luminescence time series recorded in constant light conditions of various intensities. The saturation of the solid line color increases with light intensity. (A) Blue illumination; (B) Green illumination. R.L.U. : Relative luminescence unit.

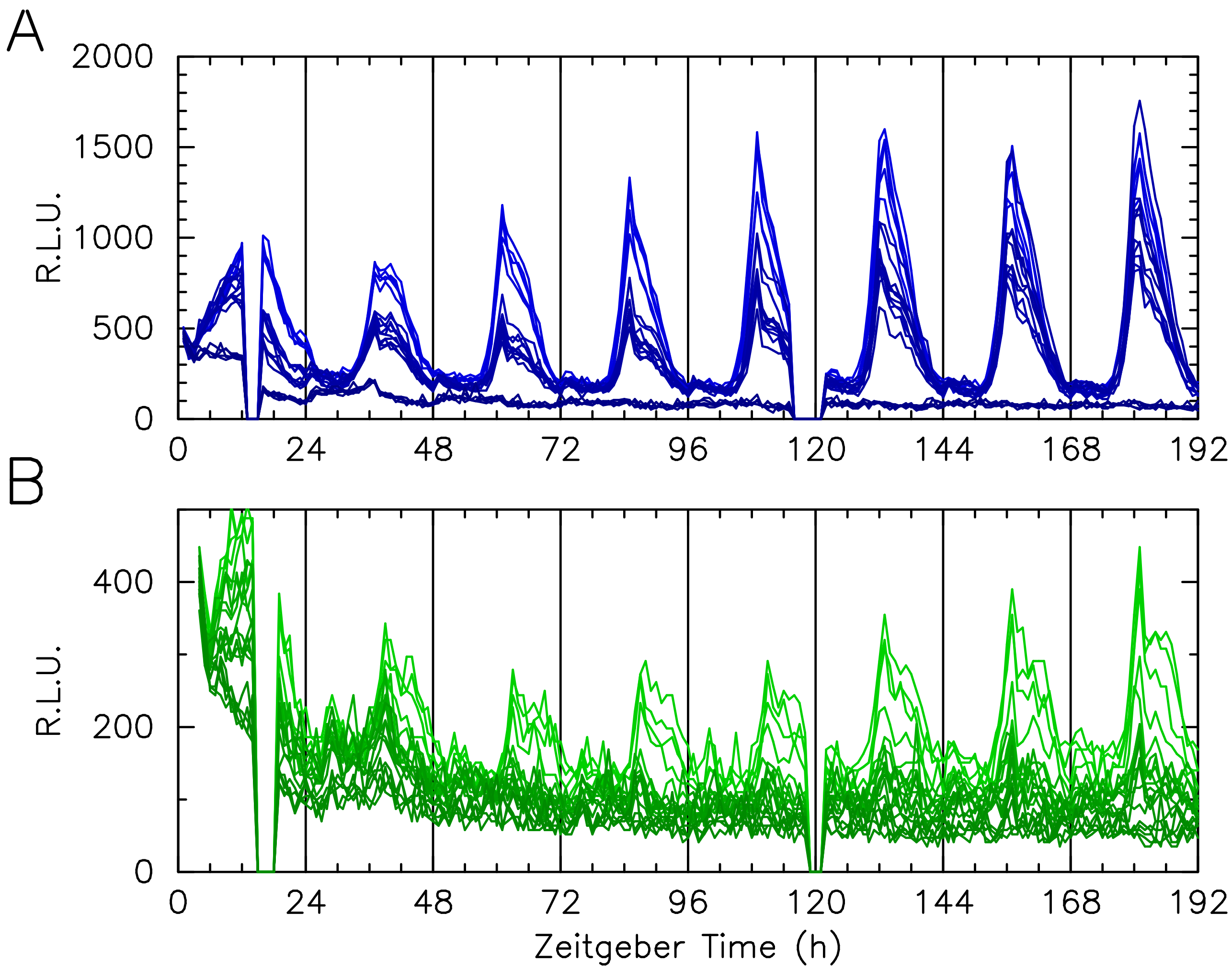

Supplementary Figure 5. Experimental time series TOC1:luc luminescence time series recorded under LD 12:12 cycles of various intensities. The saturation of the solid line color increases with light intensity. (A) Blue illumination; (B) Green illumination. R.L.U. : Relative luminescence unit.

REFERENCES

1 .Djouani-Tahri E, Christie J, Sanchez-Ferandin S, Sanchez F, Bouget F, Corellou F. A eukaryotic lov-histidine kinase with circadian clock function in the picoalga *ostreococcus*. *Plant Cell* **65** (2011) 578–88.
